# Effect of air abrasive polishing on nickel release, stainless steel corrosion, and nickel-titanium archwires

**DOI:** 10.34172/joddd.2023.40536

**Published:** 2023-12-30

**Authors:** Mohanad Ali Mohammed, Alan Issa Saleem

**Affiliations:** Department of Orthodontics, College of Dentistry, University of Baghdad, Bab Al-Muadham Campus, Baghdad, Iraq

**Keywords:** Abrasive polishing, Archwires, Nickle, Stainless steel, Titanium

## Abstract

**Background.:**

Orthodontic treatment is becoming more and more popular. However, using fixed orthodontic devices for treatment affects oral hygiene and raises the risk of corrosion, plaque-related illnesses, and dental discoloration-related issues. Air abrasive polishing has a superior effect over the conventional method in removing dental deposits. Using fixed orthodontic appliances affects oral hygiene and raises the risk of diseases caused by plaque, tooth discoloration, and corrosion, as well as corrosion by ions. This study evaluated the impact of air polishing on nickel ion release and corrosion from stainless steel, nickel-titanium, coated stainless steel, and coated nickel-titanium.

**Methods.:**

A total of 288 (stainless steel, coated stainless-steel, nickel-titanium, and coated nickel-titanium rectangular archwires) of one brand were subjected to varying air abrasion polishing times (5, 10, and 20 seconds). Then, they were submerged in artificial saliva with a pH of 6.75 and incubated for 28 days at 37 °C. The release of nickel ions (Ni^2+^) was measured using an atomic absorption spectrophotometer at 7, 14, and 28 days to estimate the cumulative effect. The corrosion of the test-selected samples and surface alterations was evaluated using scanning electron microscopy (SEM).

**Results.:**

Prolonged polishing significantly increased Ni^2+^ release and corrosion. Archwires made of coated stainless steel exhibited the least amount of Ni^2+^ release.

**Conclusion.:**

The air polishing process increased the Ni^2+^ release at a subtoxic level and could be used on adult patients with long gaps between visits with a polishing period of 5 seconds.

## Introduction

 The need for fixed orthodontic appliances has increased. However, there might be several drawbacks during orthodontic treatment, like plaque-related allergies and conditions. The unfavorable oral environment, characterized by the presence of bacteria and other microorganisms, can act as a favorable medium for the electrochemical corrosion of embedded metallic objects such as braces, wires, and accessories because the microorganisms present in the oral cavity can produce electrolytes and organic acids that can corrode the metal. In addition, the high pH levels in the oral environment can increase the corrosion rate.^1^ Furthermore, metal orthodontic components may experience increased metallic corrosion when exposed to harmful physical and chemical contaminants.^2^ Investigations into the potential mutagenic, allergenic, and carcinogenic effects of the released ions from the corrosion process of the metallic alloys used to create orthodontic wires have primarily concentrated on corrosion and the generation of corrosion byproducts (ions). According to several studies,^1,3-6^ nickel (Ni), iron (Fe), chromium (Cr), manganese (Mn), and nickel from nickel-titanium alloy are the main corrosion products for stainless steel and titanium alloys. On the other hand, fixed braces with wires interfere with thorough cleaning methods, encourage plaque build-up, and exacerbate tooth discoloration.^7^ The effectiveness of air-polishing systems in removing dental plaque has been widely studied and documented. These systems utilize abrasive particles such as calcium, sodium, silicate phosphor, sodium bicarbonate, or calcium carbonate to remove plaque from the teeth effectively. In addition to their ability to remove plaque, these systems also release controlled air and water jets, further aiding in plaque removal. Compared to traditional professional dental prophylaxis (PDP), air-polishing systems are more effective in removing plaque and require less working time and operator effort, making them an attractive option for patients and dental professionals.^8,9^

 Previous studies have shown that air polishing is the most effective and successful method for removing plaque around orthodontic brackets and archwires.^10^ This is because air polishing is more effective at eliminating stain and plaque deposits than traditional scaling and rubber cup polishing. With air polishing, the dental expert can remove stains quickly and with less effort. It has been demonstrated that using air polishers on surfaces made of enamel is safe and does not cause the enamel to slough subsequently.^11^

 Air polishing may cause gingival bleeding and abrasion, but clinically, these effects are irrelevant because they are transient.^11^ In addition to removing plaque, air polishing can also help prevent corrosion and breakdown of materials in orthodontic appliances caused by the aggressive electrolytic environment in the human mouth.^12^ The wet environment in the oral cavity contributes to electrolytic or electrochemical corrosion, which can compromise the integrity of orthodontic attachments over time.

 A surface oxide layer is created when the surface of some metals reacts with oxygen, preventing an attacking chemical from accessing the metal surface. When a metal is shielded from the elements, its ability to corrode depends on the properties of the protective covering. As long as the surface oxide layer is there, metallic materials are resistant to corrosion. However, the oxide layer dissolves when an alloy reaches its breakdown potential, which starts surface corrosion and pitting.^13^ Due to their imperfect smoothness, orthodontic wires and brackets are the most susceptible to pitting corrosion. They can have a lot of pits when viewed in microscopic detail. Because of their ability to retain bacteria that generate plaque, this property is thought to increase their susceptibility to corrosion. These microorganisms cause localized oxygen deprivation and pH decline, both of which affect the passivation process.^14,15^

 Corrosion is an electrochemical process clinically described as a loss of metal or its transformation into an oxide. Since it is an oxidative reaction, it takes place at the system’s anode. A related cathode reaction is necessary to maintain electroneutrality, and the corrosion process halts if either the anode reaction or the cathode reaction is hindered. Orthodontic appliance corrosion can have serious clinical effects, such as dimension loss that results in less force being applied to the teeth or stress corrosion failure of the appliance.^16^ The clinical significance of corrosion is numerous.^17^ Firstly, corrosion increases the frictional force at the archwire‒bracket interface by increasing surface roughness. Secondly, local pain or swelling near orthodontic appliances has been linked to corrosion products in the absence of an infection, which can result in a secondary infection. Thirdly, corrosion has a cytotoxic effect and exhibits biological reactions. Lastly, it weakens the appliance. Teflon, epoxy, polymer, and rhodium compounds, among others, are frequently used by manufacturers to coat stainless steel or nickel-titanium wires. The mechanical and frictional characteristics of archwires are likely to be affected by the presence of a coating layer. As a result, the producers always strive to coat the wires with a substance that exhibits ideal visual and frictional properties.^18^

 The present study investigated the effect of air abrasive polishing on nickel release and corrosion of stainless steel and nickel-titanium archwires.

## Methods

###  Sources of Materials

 One brand of orthodontic archwire (Ultimate Wire^TM^) was obtained from International Orthodontic Services, Stafford, USA. Stainless steel, coated stainless steel, nickel-titanium, and coated nickel-titanium rectangular archwires of one brand (The Ultimate Wire^TM^) were obtained from IOS (International Orthodontic Services, Stafford, USA).

###  Experimental Design

**Figure 1:**
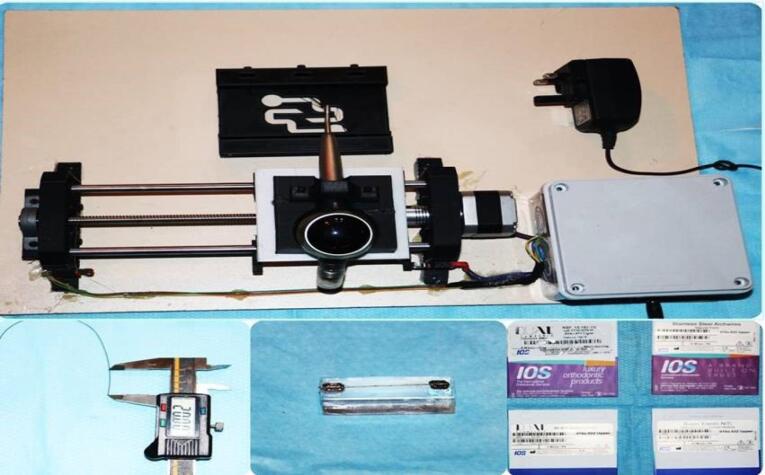
A customized holding device for air polishing, length of wire used, CNC block with wire attached to it, and archwires used

 The experiment conducted in the context above sought to comprehensively assess the impact of air polishing on stainless steel wires used in orthodontic treatment. By dividing the wire samples into distinct groups based on varying polishing durations (5, 10, and 20 seconds) alongside a control group that underwent no polishing, the researchers could meticulously analyze the outcomes (Figure 1). Employing state-of-the-art equipment in the form of Prophy-Mate Neo flash pearl calcium carbonate airborne particles and Prophy-Mate Neo polishing tools from NSK Co.^19^

 This study’s experimental setup involved using a specialized holding mechanism equipped with brackets to secure the wires for air polishing. Following the air abrasion process, the wires were meticulously extracted from the CNC block using a Wingert plier and subjected to a brief immersion in an ethanol-filled ultrasonic machine to eliminate residual calcium carbonate particles.^20^ Subsequently, the wires were introduced into vacuum-glass tubes containing 10 mL of synthetic saliva with a pH of 6.75. These tubes were securely sealed and placed within a controlled incubator environment at 37 °C for 28 days.^21^

 The wires were swiftly relocated to another tube filled with 10 mL of artificial saliva in seven days. Once again, after another seven days, the wires found a new home in a fresh tube, still containing 10 mL of artificial saliva. This meticulous process adhered to the esteemed ISO/IEC 17025:2005 standards. The primary objective of this study was to delve into the intricate relationship between polishing times and the resulting surface roughness and microhardness of stainless steel wires used in orthodontic treatment. The findings of this investigation hold immense value for clinicians, as they shed light on the optimal air polishing parameters that can lead to enhanced treatment outcomes and heightened patient comfort. With this knowledge, clinicians can revolutionize their approach, offering patients a remarkable orthodontic experience.

###  Atomic absorption spectrophotometric analysis

 After abrasion polishing, each wire was placed individually in a plain tube containing 10 mL of artificial saliva. The samples were examined after the incubation period (7, 14, and 28 days). For calculating the Ni ion release, the technician transferred the artificial saliva to a spectrophotometer, and the quantitative analysis of nickel was performed using a flam ASS with a wavelength of 341.5 nm. The technician calculated the results according to the calibration curve of the atomic absorption spectrophotometer. The release of Ni ions at 7, 14, and 28 days was evaluated using the atomic absorption spectrophotometer.

###  Scanning electron microscopy analysis

 Analysis of the surface micromorphology of the archwires was conducted using scanning electron microscopy (SEM).

###  Statistical analysis

 In this study, ANOVA was used to compare the mean survival times of the three test groups. The least significant difference (LSD) test is a post hoc test often used after ANOVA to determine which pairwise comparisons are significant. In this study, the LSD test was used to determine which pairs of group means were significantly different from each other. The significance level was determined at *P* < 0.05, meaning any results with a P-value less than 0.05 were considered statistically significant.

## Results

**Figure 2:**
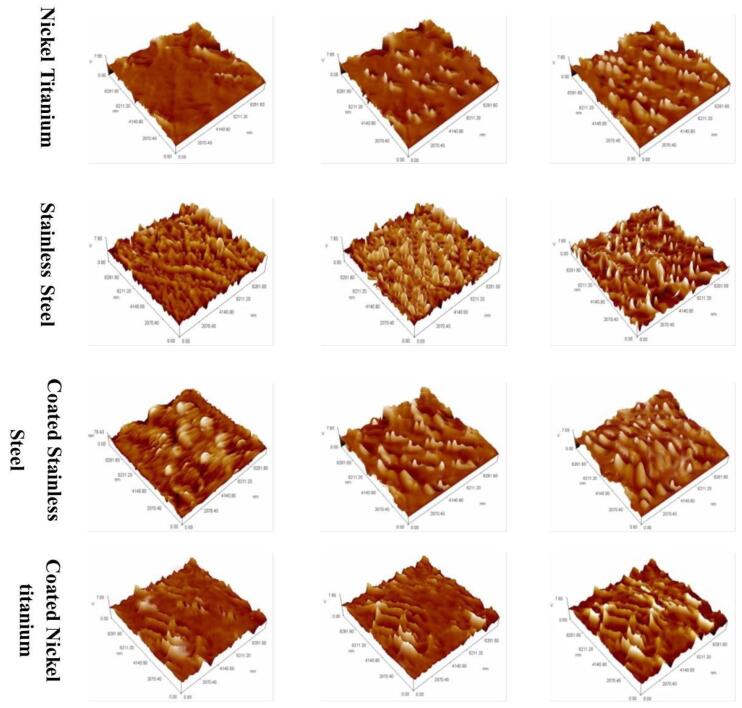
Surface micromorphology of each type of archwires at × 2000 magnification using SEM. A represents the surface of control arch wires to air polishing; B represents the surface of the arch wires after 5 seconds of air polishing; C represents the surface of archwires after 20 seconds of air polishing

 The results showed a highly significant (*P* ≤ 0.05) increase in the amount of Ni^2+^ released during the study period, coincident with an increase in polishing time. Compared to the other archwires, coated stainless steel showed minimum release types (Tables 1, 2, and 3). Before and during the application of calcium carbonate air abrasive polishing, the surface micromorphology of the archwires was analyzed and observed using SEM at a magnification of × 2000. It was discovered that the modification of the surface of the tested archwire increased with the application of air-abrasive polishing (Table 4). This was demonstrated by the emergence of multiple corroded pits of various sizes and depths concurrent with increased polishing time, as presented in Figure 2.

**Table 1:** Accumulative Ni ion release from different arch wire types at different polishing times (incubation period 7 days)

**Time of** **polishing **	**Types of wire**	**Mean** **(μg/dL)**	**SD**	**Min**	**Max**	**F-test**	***P*** ** value**
Control	Coated stainless steel	17.90817	0.103529	17.812	18.004	160.002	0.0001
	Stainless steel	18.83367	0.219093	18.632	19.035		
	Coated nickel titanium	20.22817	0.191160	20.052	20.404		
	Nickel titanium	20.44567	0.347259	20.127	20.764		
5 s	Coated stainless steel	18.13317	0.066289	18.071	18.195	122.225	0.0001
	Stainless steel	19.23267	0.241002	19.011	19.454		
	Coated nickel titanium	20.24017	0.387242	19.885	20.595		
	Nickel titanium	20.63250	0.182762	20.464	20.801		
10 s	Coated stainless steel	18.09067	0.111744	17.987	18.194	253.798	0.0001
	Stainless steel	19.17083	0.194859	18.988	19.350		
	Coated nickel titanium	20.40067	0.315491	20.111	20.690		
	Nickel titanium	20.89367	0.019765	20.874	20.913		
20 s	Coated stainless steel	19.59617	0.106172	19.498	19.701	305.901	0.0001
	Stainless steel	19.31717	0.201019	19.132	19.502		
	Coated nickel titanium	20.70950	0.026539	20.683	20.735		
	Nickel titanium	21.04983	0.055708	20.997	21.102		

**Table 2:** Accumulative Ni ion release from different arch wire types at different polishing times (incubation period14 days)

**Time of** **polishing**	**Types of wire**	**Mean** **(μg/dL)**	**SD**	**Min**	**Max**	**F-test**	***P *** **value**
Control	Coated stainless steel	18.02633	0.005502	18.021	18.032	387013.265	0.0001
	Stainless steel	18.75033	0.005502	18.745	18.756		
	Coated nickel titanium	20.68633	0.005502	20.681	20.692		
	Nickel titanium	20.81733	0.005502	20.812	20.823		
5 s	Coated stainless steel	18.81633	0.005502	18.811	18.822	222723.308	0.0001
	Stainless steel	18.79100	0.007183	18.780	18.799		
	Coated nickel titanium	20.53933	0.005502	20.534	20.545		
	Nickel titanium	20.99400	0.005477	20.989	20.999		
10 s	Coated stainless steel	19.46133	0.005502	19.456	19.467	266710.887	0.0001
	Stainless steel	19.20633	0.005502	19.201	19.212		
	Coated nickel titanium	21.17233	0.005502	21.167	21.178		
	Nickel titanium	21.47433	0.005502	21.469	21.480		
20 s	Coated stainless steel	19.83733	0.005502	19.832	19.843	249183.618	0.0001
	Stainless steel	19.69133	0.005502	19.686	19.697		
	Coated nickel titanium	21.58333	0.005502	21.578	21.589		
	Nickel titanium	21.81033	0.005502	21.805	21.816		

**Table 3:** Accumulative Ni ion release from different arch wire types at different polishing times (incubation period 28 days)

**Time of** **polishing**	**Types of wire**	**Mean** **(μg/dL)**	**SD**	**Min**	**Max**	**F-test**	***P*** ** value**
Control	Coated stainless steel	20.14467	0.004502	20.139	20.151	553229.527	0.0001
	Stainless steel	20.16067	0.004502	20.155	20.167		
	Coated nickel titanium	22.56550	0.004764	22.559	22.572		
	Nickel titanium	22.54567	0.004502	22.540	22.552		
5 s	Coated stainless steel	20.61667	0.004502	20.611	20.623	2508031.418	0.0001
	Stainless steel	20.63767	0.004502	20.632	20.644		
	Coated nickel titanium	25.75167	0.004502	25.746	25.758		
	Nickel titanium	25.35100	0.004099	25.346	25.357		
10 s	Coated stainless steel	20.86167	0.004502	20.856	20.868	3682318.034	0.0001
	Stainless steel	20.76167	0.004502	20.756	20.768		
	Coated nickel titanium	26.55100	0.004099	26.546	26.557		
	Nickel titanium	26.75100	0.004099	26.746	26.757		
20 s	Coated stainless steel	21.53767	0.004502	21.532	21.544	3946823.152	0.0001
	Stainless steel	21.53800	0.004050	21.534	21.544		
	Coated nickel titanium	27.60767	0.004502	27.602	27.614		
	Nickel titanium	27.80767	0.004502	27.802	27.814		

**Table 4:** Comparison between the mean values of Ni ions released for all different arch wire types at different polishing time

**Incubation periods**	**Time of polishing (s)**	**Types of wire**		**Mean difference**	***P *** **value**
7 days	Control	Stainless steel	Coated stainless	-0.925500^*^	0.000
			Nickel titanium	-2.320000^*^	0.000
			Coated nickel titanium	-2.537500^*^	0.000
		Coated stainless	Nickel titanium	-1.394500^*^	0.000
			Coated nickel titanium	-1.612000^*^	0.000
		Nickel titanium	Coated nickel titanium	-0.217500	0.012
	5 s	Stainless steel	Coated stainless	-1.099500^*^	0.000
			Nickel titanium	-2.107000^*^	0.000
			Coated nickel titanium	-2.499333^*^	0.000
		Coated stainless	Nickel titanium	-1.007500^*^	0.000
			Coated nickel titanium	-1.399833^*^	0.000
		Nickel titanium	Coated nickel titanium	-0.392333^*^	0.013
	10 s	Stainless steel	Coated stainless	-1.080167^*^	0.000
			Nickel titanium	-2.310000^*^	0.000
			Coated nickel titanium	-2.803000^*^	0.000
		Coated stainless	Nickel titanium	-1.229833^*^	0.000
			Coated nickel titanium	-1.722833^*^	0.000
		Nickel titanium	Coated nickel titanium	-0.493000^*^	0.000
	20 s	Stainless steel	Coated stainless	0.279000^*^	0.001
			Nickel titanium	-1.113333^*^	0.000
			Coated nickel titanium	-1.453667^*^	0.000
		Coated stainless	Nickel titanium	-1.392333^*^	0.000
			Coated nickel titanium	-1.732667^*^	0.000
		Nickel titanium	Coated nickel titanium	-0.340333^*^	0.000
14 days	Control	Stainless steel	Coated stainless	-0.724000^*^	0.000
			Nickel titanium	-2.660000^*^	0.000
			Coated nickel titanium	-2.791000^*^	0.000
		Coated stainless	Nickel titanium	-1.936000^*^	0.000
			Coated nickel titanium	-2.067000^*^	0.000
		Nickel titanium	Coated nickel titanium	-0.131000^*^	0.000
	5 s	Stainless steel	Coated stainless	0.025333^*^	0.000
			Nickel titanium	-1.723000^*^	0.000
			Coated nickel titanium	-2.177667^*^	0.000
		Coated stainless	Nickel titanium	-1.748333^*^	0.000
			Coated nickel titanium	-2.203000^*^	0.000
		Nickel titanium	Coated nickel titanium	-0.454667^*^	0.000
	10 s	Stainless steel	Coated stainless	0.255000^*^	0.000
			Nickel titanium	-1.711000^*^	0.000
			Coated nickel titanium	-2.013000^*^	0.000
		Coated stainless	Nickel titanium	-1.966000^*^	0.000
			Coated nickel titanium	-2.268000^*^	0.000
		Nickel titanium	Coated nickel titanium	-0.302000^*^	0.000
	20 s	Stainless steel	Coated stainless	0.146000^*^	0.000
			Nickel titanium	-1.746000^*^	0.000
			Coated nickel titanium	-1.973000^*^	0.000
		Coated stainless	Nickel titanium	-1.892000^*^	0.000
			Coated nickel titanium	-2.119000^*^	0.000
		Nickel titanium	Coated nickel titanium	-0.227000^*^	0.000
28 days	Control	Stainless steel	Coated stainless	-0.016000^*^	0.000
			Nickel titanium	-2.420833^*^	0.000
			Coated nickel titanium	-2.401000^*^	0.000
		Coated stainless	Nickel titanium	-2.404833^*^	0.000
			Coated nickel titanium	-2.385000^*^	0.000
		Nickel titanium	Coated nickel titanium	0.019833^*^	0.000
	5 s	Stainless steel	Coated stainless	-0.021000^*^	0.000
			Nickel titanium	-5.135000^*^	0.000
			Coated nickel titanium	-4.734333^*^	0.000
		Coated stainless	Nickel titanium	-5.114000^*^	0.000
			Coated nickel titanium	-4.713333^*^	0.000
		Nickel titanium	Coated nickel titanium	0.400667^*^	0.000
	10 s	Stainless steel	Coated stainless	0.100000^*^	0.000
			Nickel titanium	-5.689333^*^	0.000
			Coated nickel titanium	-5.889333^*^	0.000
		Coated stainless	Nickel titanium	-5.789333^*^	0.000
			Coated nickel titanium	-5.989333^*^	0.000
		Nickel titanium	Coated nickel titanium	-0.200000^*^	0.000
	20 s	Stainless steel	Coated stainless	-0.000333	0.000
			Nickel titanium	-6.070000^*^	0.000
			Coated nickel titanium	-6.270000^*^	0.000
		Coated stainless	Nickel titanium	-6.069667^*^	0.000
			Coated nickel titanium	-6.269667^*^	0.000
		Nickel titanium	Coated nickel titanium	-0.200000^*^	0.000

## Discussion

 Adult patients in need of orthodontic care are becoming more prevalent today. The eating habits of this group of patients were different from those of adolescents in that they consumed more colored liquids, such as coffee and tea, which stain the enamel and leave a deposit, necessitating a cleaning procedure.^22^ In the past, using a rubber cup or brush to apply an abrasive paste to teeth during PDP has been the standard procedure for polishing teeth. This procedure can be used to get rid of supragingival plaque and stains. However, it is difficult, time-consuming, and ineffective to remove supragingival deposits and stains from the region around bonded orthodontic appliances using a rubber cup and abrasive paste.^23^ Therefore, it can be deduced that airflow polishing offers advantages over traditional PDP despite its inability to effectively remove dental plaque and discoloration due to its encouragement of reduced working time and operator effort. Furthermore, this approach has been widely employed to address adult patients’ compromised orthodontic treatment compliance and treatment satisfaction by removing tooth discoloration.^8,9^ Dental alloys can release metal ions into the oral cavity due to corrosion processes, even though they have a protective oxide coating on the metal surface.^24^ The Ni^2+^ released from archwires after air polishing has not been previously studied. To demonstrate and emphasize how air polishing affects the Ni^2+^ release in synthetic saliva, this study performed three separate air polishing sessions using a calcium carbonate powder.^24-26^

 Previous studies on metal ions released from orthodontic archwires have found that the corrosion process can take up to four weeks to complete, suggesting that corrosion is a slow process and that it may take an extended period for the release of metal ions to reach its maximum potential. In this study, the incubation period for the archwires in synthetic saliva was set at 28 days, equivalent to approximately four weeks.^27,28^ The increase in Ni^2+^ release that happens simultaneously with an increase in polishing time may be due to the rise in the surface roughness of archwires, increasing the surface area of the wire. Longer polishing durations were predicted to lead to an increase in surface area and texture roughness.^20^ In addition, when the surface roughness increases, the surface area that comes into contact with the saliva increases, thereby increasing the Ni^2+^ released.^29^ According to Pakshir et al^30^ in 2011 and Roberge^31^ in 2012, when manufacturers create pits, the passive layer is locally dissolved, and the pit’s depth in the underlying metal increases quickly.^30,31^ As a result, these surface irregularities accelerate the corrosion process. More ions were produced and detected due to the development of an electrochemical cell in which the cathode is a sizable region of passive metal, and a very small area of active metal is the anode.^30,31^ In addition, it was reported that a passive layer rich in chromium and typically 3‒5 nm in depth, or around 15 layers of atoms, gives the material its corrosion-resistant feature.^32^

 An oxidation-reduction reaction produces the passive layer, during which the passivating substance is reduced, and chromium and nickel are oxidized. Rapid general and/or galvanic corrosion may result if this layer is not permitted to form or if it is damaged.^32^ The stainless steel and titanium alloys used in orthodontic appliances prevent corrosion by forming a passive surface oxide coating. The barrier is vulnerable to mechanical and chemical damage; hence, it is not impenetrable. SEM studies of archwires made of stainless steel and Ni-Ti subjected to electrochemical corrosion in artificial saliva by air prophy revealed pitting corrosion on the wire surface.^17^

 The polishing process of abrasive particles damages the chromium oxide layer, a protective passive layer that is likely destroyed, exposing the fresh metal to corrosion and speeding up surface degradation.^33,34^ The total amount of Ni^2+^ in each of the four major groups was lower than their combined daily intake. The results of the present study showed that the adult acceptable upper intake threshold for nickel is 45 mg/day, indicating that all discharged ions present following the air polishing professional cleaning method were below daily maximums and hazardous values.^35,36^ Metallic orthodontic appliances may release metal ions due to corrosion in the oral cavity. Airflow polishing may impact the ion release from orthodontic wires.^37^

## Conclusion

 The results of this study provide valuable insights into the potential use of calcium carbonate air polishing during orthodontic treatment. This technique can be effectively applied by adhering to the recommended polishing time of 5 seconds and implementing longer polishing pauses for adults. Furthermore, this investigation sheds light on the impact of air-powder polishing on surface roughness and topography, particularly focusing on orthodontic archwires. Notably, the use of calcium bicarbonate powder in this method has been found to alter the surface roughness and topography of the archwires, a finding that has not been previously explored in vitro. As a practical recommendation, orthodontists should consider employing new archwires after air-powder polishing, particularly when performing tooth movements requiring reduced friction. By incorporating these findings into clinical practice, orthodontists can enhance treatment outcomes and optimize patient care.

## Acknowledgments

 The authors are grateful to the College of Dentistry/ University of Mosul for providing facilities to accomplish this work.

## Competing Interests

 The authors declare no competing interests.

## Ethical Approval

 College of Dentistry/University of Baghdad** (**UoB/CoD 600 on 10.04.2022).

## Funding

 Self-funded.
